# Advance directives in patients with head and neck cancer - status quo and factors influencing their creation

**DOI:** 10.1186/s12904-022-00932-5

**Published:** 2022-04-08

**Authors:** Moritz Allner, Magdalena Gostian, Matthias Balk, Robin Rupp, Clarissa Allner, Konstantinos Mantsopoulos, Christoph Ostgathe, Heinrich Iro, Markus Hecht, Antoniu-Oreste Gostian

**Affiliations:** 1 Department of Otorhinolaryngology, Head & Neck Surgery, Comprehensive Cancer Center Erlangen-EMN, Friedrich-Alexander-Universität (FAU), 91054 Erlangen-Nürnberg, Germany; 2 Department of Anesthesiology, Malteser Waldkrankenhaus St. Marien, Erlangen, Germany; 3 Emergency Medical Center, Department of Internal Medicine, Klinikum Fürth, Fürth, Germany; 4 Department of Palliative Medicine, Comprehensive Cancer Center Erlangen-EMN, Friedrich-Alexander-Universität (FAU), Erlangen-Nürnberg, Germany; 5 Department of Radiation Oncology, Comprehensive Cancer Center Erlangen-EMN, Friedrich-Alexander-Universität (FAU), Erlangen-Nürnberg, Germany

**Keywords:** Advance care planning, Advance directive, Head and neck Cancer, Living will, Patient autonomy, Power of attorney for healthcare

## Abstract

**Background:**

Advance Care Planning including living wills and durable powers of attorney for healthcare is a highly relevant topic aiming to increase patient autonomy and reduce medical overtreatment. Data from patients with head and neck cancer (HNC) are not currently available. The main objective of this study was to survey the frequency of advance directives (AD) in patients with head and neck cancer.

**Methods:**

In this single center cross-sectional study, we evaluated patients during their regular follow-up consultations at Germany’s largest tertiary referral center for head and neck cancer, regarding the frequency, characteristics, and influencing factors for the creation of advance directives using a questionnaire tailored to our cohort. The advance directives included living wills, durable powers of attorney for healthcare, and combined directives.

**Results:**

Four hundred and forty-six patients were surveyed from 07/01/2019 to 12/31/2019 (response rate = 68.9%). The mean age was 62.4 years (SD 11.9), 26.9% were women (*n* = 120). 46.4% of patients (*n* = 207) reported having authored at least one advance directive. These documents included 16 durable powers of attorney for healthcare (3.6%), 75 living wills (16.8%), and 116 combined directives (26.0%). In multivariate regression analysis, older age (OR ≤ 0.396, 95% CI 0.181–0.868; *p* = 0.021), regular medication (OR = 1.896, 95% CI 1.029–3.494; *p* = 0.040), and the marital status (“married”: OR = 2.574, 95% CI 1.142–5.802; *p* = 0.023; and “permanent partnership”: OR = 6.900, 95% CI 1.312–36.295; p = 0.023) emerged as significant factors increasing the likelihood of having an advance directive. In contrast, the stage of disease, the therapeutic regimen, the ECOG status, and the time from initial diagnosis did not correlate with the presence of any type of advance directive. Ninety-one patients (44%) with advance directives created their documents before the initial diagnoses of head and neck cancer. Most patients who decide to draw up an advance directive make the decision themselves or are motivated to do so by their immediate environment. Only 7% of patients (*n* = 16) actively made a conscious decision not create an advance directive.

**Conclusion:**

Less than half of head and neck cancer patients had created an advance directive, and very few patients have made a conscious decision not to do so. Older and comorbid patients who were married or in a permanent partnership had a higher likelihood of having an appropriate document. Advance directives are an essential component in enhancing patient autonomy and allow patients to be treated according to their wishes even when they are unable to consent. Therefore, maximum efforts are advocated to increase the prevalence of advance directives, especially in head and neck cancer patients, whose disease often takes a crisis-like course.

**Supplementary Information:**

The online version contains supplementary material available at 10.1186/s12904-022-00932-5.

## Introduction

The incidence of head and neck cancer (HNC) is continuously increasing and now ranks seventh among all newly diagnosed malignancies worldwide [1]. The 5-year survival rate has long remained unchanged at between only 40 and 50% [2]. Alt-Epping et al. pointed out that compared with other cancer entities or other non-oncologic underlying conditions, patients with HNC are often associated with crises during the course of disease and suffer from local complications, such as difficulties with tracheostomy, dyspnea, and even anaphylactic conditions or fatal mass hemorrhage. In addition, temporary or even permanent therapeutic measures, such as a tracheostomy, further limit the ability to communicate. Additionally, the proportion of older, multimorbid patients with HNC is increasing, leading to an even higher risk of life-threatening complications [3–5]. Lori J. Bernstein et al. also demonstrated in their 2018 study that survivors of HNC are at increased risk for neurocognitive sequelae up to 2 years after definitive chemotherapy or radiation therapy. Accordingly, patients with HNC are also more likely to have impaired ability to consent as the disease progresses [6]. This aspect should be considered and included in head and neck oncology treatment planning.

Next to the medical indication, the patient’s wishes are the primary factors protecting patient autonomy for any medical intervention. An advance directive (AD) covers various types of documents created to describe the extent of medical treatment a patient wants - or does not want - to receive. In the event that patients lose the ability to consent, they can state their preferences for certain future medical conditions in a living will (LW) [7, 8]. With a durable power of attorney for healthcare (DPAHC), patients can authorize a trusted person to represent their probable wishes by proxy. Combined directives (CDs) are considered useful and desirable [9]. They can ensure that the patient’s wishes are implemented, if necessary, even against any resistance to the contents of the LW. In this publication, an AD is defined as the presence of either a LW, a DPAHC or a CD.

The number of ADs in Germany has risen significantly in recent years, especially after changes in the law on ADs [Drittes Gesetz zur Änderung des Betreuungsrechts] in 2009 [10, 11]. The aim was to create a secure legal framework for documenting the patient’s wishes [12]. According to a survey of the German Hospice and Palliative Care Association, the proportion of people possessing an LW/DPAHC has increased from 26 to 43% since 2012 [13]. There is also a substantially growing need for professional counseling and support in creating and writing an LW/DPAHC. In 2017, the German Foundation for Patient Protection reported the highest level of counseling to date, with an increase of 13% over the previous year [10].

Data on the frequency of ADs (0–51%) vary widely in international comparisons and in relation to different patient populations [14–18]. Studies specifically on cancer patients showed LW prevalences of between 0 and 41% [19, 20]. Data from clinical practice in HNC patients are delivered not currently available.

Despite the relevance of the topic, there are currently not enough studies to determine the frequency and applicability of ADs especially in patients with HNC. Therefore, the main objective of this study was to survey the frequency of an LW/DPAHC/CD in patients with HNC. Further objectives included the identification of relevant clinical and sociodemographic factors influencing the creation and availability of ADs. We also asked patients about the timing of the creation of their ADs and the reasons for and against their creation.

## Material and methods

In this prospective single center study, patients were asked about their ADs during the regular cancer follow-up visits at Germany’s largest tertiary referral center for HNC at the Department of Otorhinolaryngology, Head and Neck Surgery, Erlangen University Hospital, between July 1, 2019 and December 31, 2019. The study was conducted according to the Declaration of Helsinki, approved by the Ethics Committee of the University of Erlangen (No.: 76_19 B), and was registered in the German Registry for Clinical Studies (DRKS) (application No.: 00017123). An AD was defined as a living will (LW), a durable power of attorney for healthcare (DPAHC), or a document combining the two – a combined directive (CD). Inclusion criteria for participation were as follows: former or current diagnosis of HNC of any tumor stage according to the Union international contre le cancer (UICC) classification, age ≥ 18 years, sufficient cognitive and language skills to answer the questionnaire independently, having answered the relevant questions of the questionnaire ([1] Does an AD exist? [2] Was it delivered to the hospital?). All patients gave their written informed consent to participate. The following exclusion criteria were applied: lack of a diagnosis of HNC, cognitive impairment, or refusal to participate in the study.

During the survey period, a total of 30 questionnaires were prepared each week and handed out to patients who had previously verbally agreed to participate, until all questionnaires were distributed. The order in which the patients appeared for the consultation and were consequently included, was random. Patients were asked to fill out a standardized two-sided questionnaire according to de Heer et al. [14]. However, question #4 (“Mode of admission?”) was removed because it was irrelevant to the present study (see Supplement 1). In the questionnaire, patients were asked whether they possessed an LW and/or DPAHC and, if so, about their motivation to create the corresponding document and about the time at which they had created it. We included a total of 446 patients that filled out the relevant questions within the questionnaire. Therefore, a total of 88 patients were excluded because of insufficiently completed questionnaires. Patients were also asked about sociodemographic factors like age, education level, family status, living environment and religion. We divided the patients into five age groups. Regarding the educational level reasonable data was evaluable from 243 patients and were divided into academic and non-academic professions for analysis. Patients who did not indicate an occupation or who answered “retired” were excluded. We derived the factor “religiosity” from the data on religious affiliations. Atheist patients were compared with other religions. In addition to the questionnaire, clinical and oncological characteristics (e.g. medication, cancer location, the presence of synchronous or metachronous secondary cancer, the time from initial diagnosis to survey period and/or locoregional or distant metastases) were obtained from the patient’s medical records. Pre-existing comorbidities were classified according to medical disciplines (cardiovascular, pulmonary, neurologic, oncologic). Tumors were staged using the 8th edition of the Tumor Node Metastasis (TNM) and the UICC classifications [21] and divided into 6 groups for better overview (oral cavity and oropharynx; hypopharynx and larynx; nose and nasopharynx; salivary glands; other HNC entities, i.e. skin, outer ear or CUP-syndrome; multi-tier carcinoma where origin of the tumor was not identifiable). At the time of the survey, we retrospectively documented treatment modality (surgical treatment, primary surgical treatment with adjuvant therapy, definitive radiochemotherapy, salvage surgery, and the current ECOG status from the patient’s records [22]. (Table 1) shows all the criteria considered. Unfortunately, we were only able to collect all relevant influencing factors from the patient record for 339 patients. Therefore, the patient collective was reduced by a further 107 patients.

**Table 1:** Patient characteristics

Variable	N (n_**total**_ = 446)	%
Gender	446	100
Female	120	26.9
Male	326	73.1
Age in years (Mean ± SD)	62.4 ± 11.9	
≤ 30	6	1.3
31–45	22	4.9
46–65	243	54.5
66–75	109	24.4
> 75	66	14.8
Marital status	436	97.8
Single	55	12.3
Married	298	66.8
Permanent partnership	13	2.9
Divorced	35	7.8
Widowed	35	7.8
Educational level	243	54.5
Non-academic	195	43.7
Academic	48	10.8
Comorbidities	446	
None	95	21.0
At least one comorbidity	351	79.0
Cardiovascular comorbidity	176	39.6
Pulmonal comorbidity	63	14.2
Neurological comorbidity	47	10.6
Oncological comorbidity	99	22.3
Regular medication	401	89.9
Yes	287	64.3
No	114	25.6
Living Environment	411	92.2
Independent at home	357	80.0
At home with support	49	11.0
Care facility	5	1.2
Religion	413	92.6
Protestant	182	40.8
Roman-catholic	173	38.8
Muslim	5	1.1
Other religion	27	6.5
Atheist	26	5.8
Religiosity	413	92.6
Yes	387	86.8
No	26	5.8
Cancer location	446	100
Oral cavity & oropharynx	147	33.0
Hypopharynx & larynx	116	26.0
Nose & nasopharynx	34	7.6
Salivary glands	61	13.7
Other head and neck cancer entities	72	16.1
Multi-tier carcinoma	16	3.6
Secondary malignancies^a^	432	96.9
No	360	80.7
Yes, synchronous	18	4.0
Yes, metachronous	54	12.2
Recurrence of cancer	437	98.0
No	395	88.6
Loco-regional recurrence	39	8.7
Distant metastases	3	0.7
UICC stadium	385	86.3
0	6	1.3
I	132	29.6
II	67	15.0
III	61	13.7
IV	119	26.7
ECOG performance status	386	86.5
0	289	64.8
1	74	16.6
2	21	4.7
3	2	0.4
Applied therapy	446	100
Surgery	171	38.3
Primary surgery plus adjuvant therapy	71	15.9
Definitive radiochemotherapy	198	44.4
Salvage surgery	6	1.3
Time between initial diagnosis and survey period		
0–12 months	113	25.3
13–60 months	178	39.9
61–120 months	92	20.6
120+ months	63	14.1

^a^Also outside the head and neck area

The frequency of ADs in our study population was defined as the primary endpoint. Secondary endpoints included clinical, therapeutic, and sociodemographic factors, as well as the factors influencing the creation of an AD and the timing and patient motivation regarding the creation of ADs.

### Statistical analysis

Categorical variables were reported as absolute frequencies (n) with percentages (%) and continuous variables as mean (M) with standard deviation (SD). To identify significant differences between the presence of ADs within sociodemographic, clinical, and oncological factors, all factors were pretested using univariate logistic regression (Table 2). The relevant variables identified in this way were analyzed for their influence on the presence of ADs using multivariate logistic regression. The age group over 75 years served as a reference category, since it is known from the literature that the presence of ADs in other patient populations is associated with a higher age [14, 18, 19, 23]. For marital status, we chose “single” and for the educational level we chose “non-academic” as the reference. For pre-existing comorbidities, medication, religiosity, secondary malignancies, and recurrence of cancer we selected “no” as the reference answer. We chose stage “IV” in the UICC-category and functional status “0” in the ECOG category, because those were the most frequent answers. For cancer locations, oral cavity- and oropharyngeal carcinomas were selected as reference. And for the time between initial diagnosis and interview period, we chose the longest period (> 10 years) as the reference category. For the therapy modality, we chose “surgery only” as the reference. Only those data sets were considered in which values were available for all the variables concerned (“listwise deletion of missing cases”). Patients who did not know whether they had AD were also not included in the analysis. A total of 339 patients were included in the multivariate regression analysis. The flow diagram displays the recruitment- and exclusion process (Supplement 3).

**Table 2:** Influencing Factors– Univariate Logistic Regression Analysis

Variable	N	OR	95% CI	Result of statistical analysis (***p***-value)
Gender (Female, *n* = 120)	446			
Male	326	1.081	[0.710–1.646]	0.717
Age group (> 75, *n* = 66)	446			
≤ 30	6	0.000	[0.000–0.000]	.
31–45*	**22**	**0.059**	**[0.016–0.224]**	< 0.001*
46–65*	**243**	**0.253**	**[0.139–0.461]**	< 0.001*
66–75*	**109**	**0.426**	**[0.221–0.825]**	0.011*
Marital status (Single, *n* = 55)	436			
Married*	298	**4.221**	**[2.099–8.488]**	< 0.001*
Divorced*	**13**	**3.000**	**[1.171–7.684]**	0.022*
Widowed*	**35**	**3.778**	**[1.482–9.631]**	0.005*
Permanent partnership*	13	**6.400**	**[**1.748–23.438**]**	0.005*
Education level (Academic, *n* = 48)	243	1.155	[0.607–2.200]	0.661
At least one comorbidity (Yes)	**401**	**2.372**	**[1.501–3.750]**	0.001*
Cardiovascular comorbidity (Yes)	**446**	**1.593**	**[1.087–2.334]**	0.017*
Pulmonal comorbidity (Yes)	446	1.058	[0.620–1.804]	0.836
Neurological comorbidity (Yes)	446	1.232	[0.673–2.255]	0.500
Oncological comorbidity (Yes)	446	1.235	[0.790–1.931]	0.355
Regular medication (Yes)*	**401**	**2.372**	**[1.501–3.750]**	< 0.001*
Living environment (Living independent at home, *n* = 357)	411			
At home with support	49	1.172	[0.645–2.130]	0.603
Care facility	5	0.281	[0.031–2.541]	0.259
Religion (Protestant, *n* = 182)	413			
Roman-catholic	173	0.752	[0.495–1.141]	0.181
Muslim	5	0.000	[0.000–0.000]	0.999
Other religion	27	0.744	[0.508–2.581]	0.744
Atheist	26	0.672	[0.293–1.541]	0.348
Religiosity (Yes)	387	1.288	[0.577–2.876]	0.536
Cancer location (oral cavity & oropharynx, *n* = 147)	446			
Hypopharynx & larynx	116	1.069	[0.656–1.742]	0.789
Nose & nasopharynx	34	0.760	[0.354–1.631]	0.481
Salivary glands	61	1.446	[0.794–2.634]	0.227
Other head and neck cancer entities	72	1.227	[0.686–2.159]	0.477
Multi-tier carcinoma	16	0.558	[0.185–1.686]	0.301
Secondary malignancies^a^ (No, *n* = 360)	432			
Yes, synchronous	18	0.827	[0.321–2.133]	0.695
Yes, metachronous	54	0.662	[0.372–1.177]	0.160
Recurrence of cancer (No, *n* = 395)	437			
Loco-regional recurrence	39	1.003	[0.519–1.941]	0.993
Distant metastases	3	0.585	[0.053–6.506]	0.663
UICC stadium (Stadium IV, *n* = 119)	385			
0	6	0.492	[0.087–2.787]	0.423
I	132	0.702	[0.426–1.157]	0.165
II	67	0.797	[0.437–1.454]	0.460
III	61	0.834	[0.449–1.549]	0.566
ECOG performance status (Stadium 0, *n* = 289)	386			
1	74	1.255	[0.753–2.093]	0.383
2	**21**	**2.973**	**[1.122–7.880]**	0.028*
3	2	1.189	[0.074–19.200]	0.903
Applied therapy (Surgery, *n* = 171)	446			
Primary surgery plus adjuvant therapy	198	1.168	[0.775–1.761]	0.497
Definitive radiochemotherapy	71	0.823	[0.470–1.442]	0.457
Salvage surgery	6	0.596	[0.106–3.342]	0.556
Time between initial diagnosis and survey period (120+ months, *n* = 63)	446			
0–12 months	113	1.104	[0.594–2.051]	0.754
13–60 months	178	1.020	[0.572–1.819]	0.945
61–120 months	92	1.250	[0.657–2.379]	0.497

Reference categories: age group (> 75 years), marital status (single), educational level (non-academic) at least one comorbidity (no), cardiovascular comorbidity (no), Pulmonal comorbidity (no), neurological comorbidity (no), oncological comorbidity (no), regular medication (no), living environment (living independent at home), religion (Protestant), religiosity (no), Cancer location (oral cavity & oropharynx), secondary malignancy (no), recurrence of cancer (no), UICC stadium (IV), ECOG performance status (0), applied therapy (surgery), time between initial diagnosis and survey period (120+ months)

Rows marked with an asterisk (*) indicate the influencing factors that were found to be statistically relevant

*N* Number of patients, *OR* Odds ratio, *95% CI* 95% confidence interval, *p* value of significance

^a^Also outside the head and neck area

Results with a *p*-value of ≤0.05 were considered significant and marked with an asterisk (*). All analyses were performed using SPSS Statistics version 28.0 software (IBM, New York, USA).

## Results

During the study period, a total of 30 patients per week were invited to participate by staff members. Thus, the study questionnaires were handed out to all cancer patients, regardless of patient characteristics throughout the respective day. A total of 775 questionnaires were distributed during the study period, of which we received 534 questionnaires back, corresponding to a response rate of 68.9%. Of these, 446 patients met the inclusion criteria (83.5%) and were included in the following analysis.

### Patient characteristics

(Table 1) presents the characteristics of all patients included. Among the 446 patients (mean age 62.4 years, SD 11.9), 120 were women (26.9%; mean age 61.5 years, SD 13.5) and 326 were men (73.1%; mean age 62.8 years, SD 11.2). Only 12.6% of patients were single, while the majority were currently or had been in a partnership. Two-thirds of them were married (68.3%; *n* = 298) or living in a permanent partnership (3.0%; *n* = 13). 70 patients were either widowed (8.0%; *n* = 35) or divorced (8.0%; *n* = 35). 357 patients (86.9%) lived independently at home, and only a minority were living in a care facility (1.2%; *n* = 5) or at home with support (11.9%; *n* = 49). Most patients (79%; *n* = 351) had at least one comorbidity and had to take medication on a regular basis (64.3%; *n* = 287). 46.7% of patients had a UICC stage III or higher and the most common cancer sites were oral cavity and oropharyngeal carcinomas (33.0%; *n* = 147) and hypopharyngeal and laryngeal carcinomas (26.0%; *n* = 116). The mean time since diagnosis was 45.6 months (SD 44.5; median = 32). Eighteen patients (4.2%) were diagnosed with synchronous and 54 patients (12.5%) with metachronous secondary malignancies. 38.3% of patients had undergone surgical treatment alone, while another 44.4% had received additional adjuvant therapy, i.e., radiotherapy (RT) or radiochemotherapy (RCT). Primary definitive concurrent radiochemotherapy (pRCT) was given in 15.9% of cases. Only 1.3% underwent salvage surgery. A locoregional recurrence of cancer occurred in 39 cases (8.9%) and distant metastasis in 3 cases (0.7%). 94% of patients surveyed had an ECOG status of 0 or 1.

### Frequency and factors influencing the presence of ADs

**Fig. 1:**
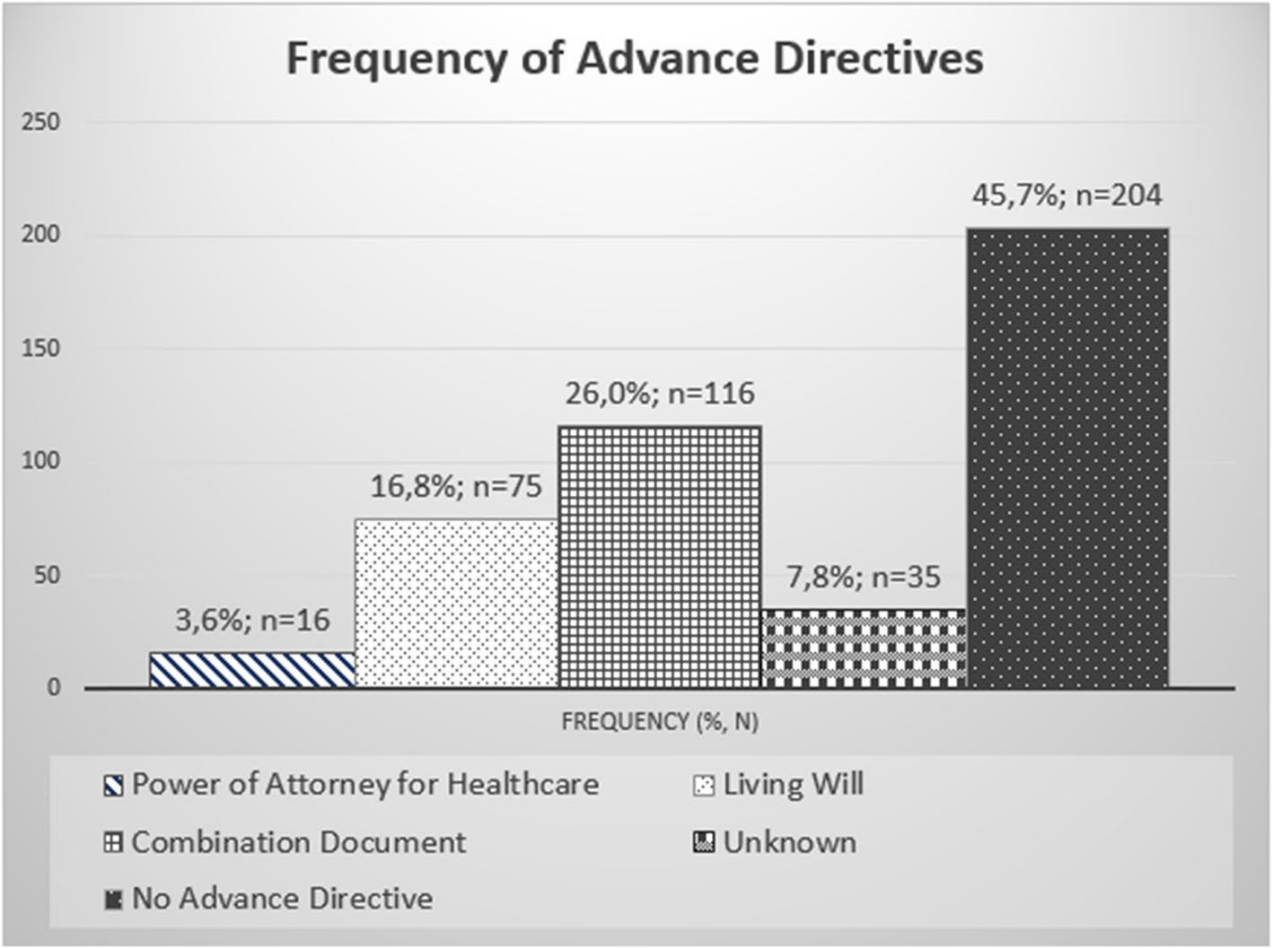
Presence of the different types of advance directives

**Fig. 2:**
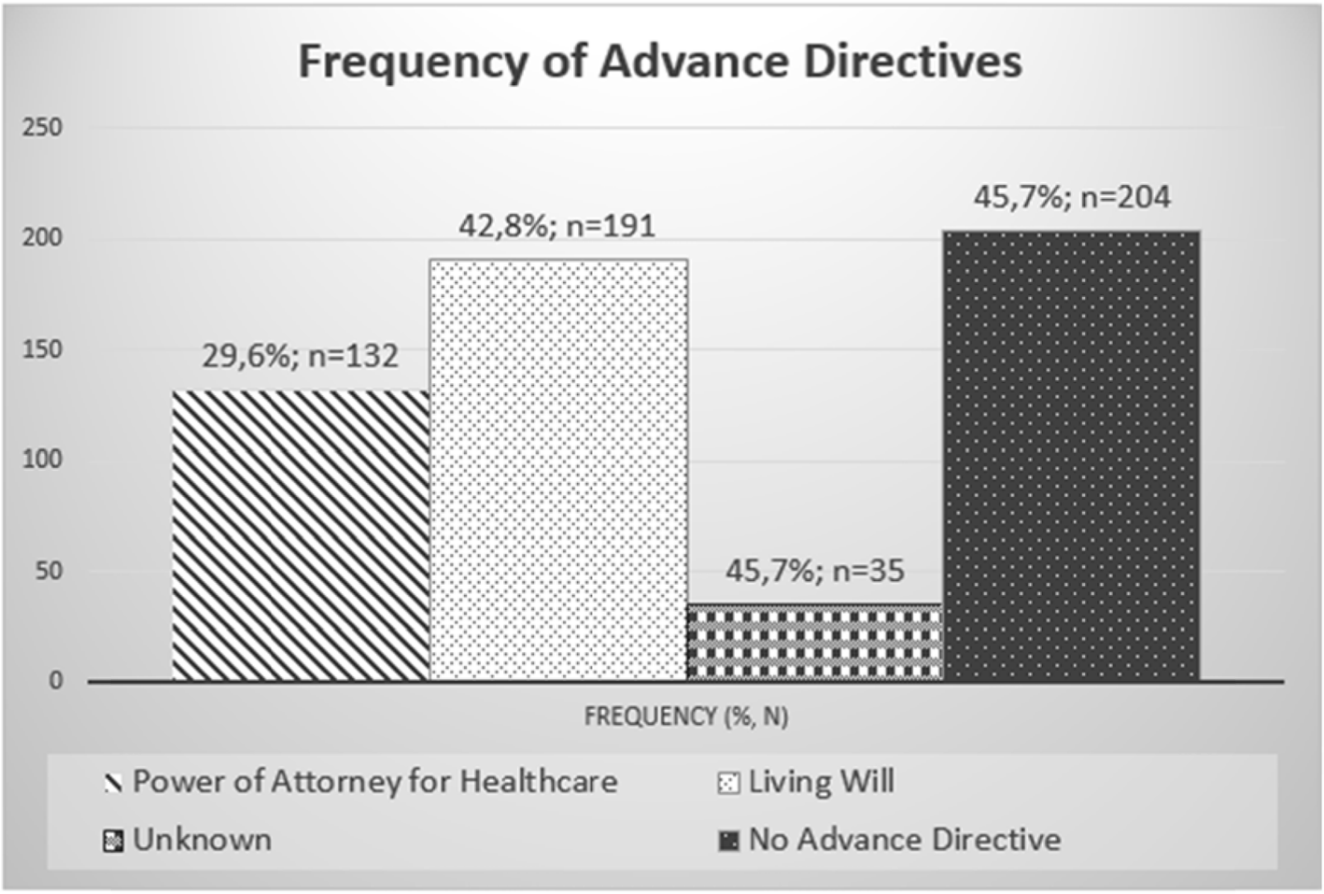
Presence of the different types of advance directives, taking LWs and DPAHCs separately

Out of the 446 patients included, 207 patients (46.4%) had at least one AD. These included 16 patients with a DPAHC (3.6%), 75 patients (16.8%) with an LW, and 116 patients with a CD (26.0%). Thirty-five patients (7.8%) did not know whether they had an AD, while 204 patients (45.7%) certainly did not have one. Considering DPAHCs and LWs separately – since CDs always contain both – DPAHCs existed in 29.6% (*n* = 132) and LWs in 42.8% (*n* = 191) of the cases. The results are shown in (Figs. 1 and 2).

Univariate analysis revealed six significant influencing factors (including age, marital status, pre-existing comorbidities and ECOG status) with a significantly higher likelihood of having at least one AD. As age increased, so did the likelihood of having an AD. No patient younger than or equal to 30 years had any AD at all. Patients between 31 and 45 years of age were less likely to have ADs than patients over 75 (odds ratio [OR] = 0.059, 95% confidence interval [95% CI] 0.016–0.224; *p* < 0.001). Compared with patients over 75 years of age, both, patients aged 46–65 years (OR = 0.253, 95% CI 0.139–0.461; *p* < 0.001) and 66–75 years were (OR = 0.426, 95% CI 0.221–0.825; *p* = 0.011), were less likely to have ADs, respectively.

Family status also had an impact on the existence of ADs, as patients living in a partnership had significantly more ADs than those living alone. Married patients were more likely to have an AD than singles (OR = 4.221, 95% CI 2.099–8.488; *p* < 0.001). Patients living in a permanent partnership were also significantly more likely to have an AD compared with single patients (OR = 6.400, 95% CI 1.748–23.438; *p* = 0.005). Divorced (OR = 3.000, 95% CI 1.171–7.684; *p* = 0.022) and widowed patients (OR = 3.778, 95% CI 1.482–9.631; *p* = 0.005) also had ADs more often than singles.

Patients with “at least one current comorbidity” (OR = 2.372, 95% CI 1.501–3.750; *p* = 0.001) or a “cardiovascular comorbidity” (OR = 1.593, 95% CI 1.087–2.334; *p* = 0.017) were significantly more likely to have ADs. Patients on “regular medication” (OR = 2.372, 95% CI 1.501–3.750; *p* < 0.001) had created ADs significantly more often.

Patients with higher ECOG functional status (2) were more likely to have ADs compared with patients with ECOG 0 (OR = 2.973, 95% CI 1.122–7.880; *p* = 0.028) However, a significant result was shown only between ECOG 0 and ECOG 2. This may be due to the small case number of patients with ECOG greater than or equal to 3 (*n* = 2).

In the multivariate approach, the factors “regular medication” (OR = 1.896, 95% CI 1.029–3.494; *p* = 0.040), “marital status” (“married”: OR = 2.574, 95% CI 1.142–5.802; *p* = 0.023 or “permanent partnership”: OR = 6.900, 95% CI 1.312–36.295; *p* = 0.023) and “higher age” correlated significantly with the presence of an AD. The older the patient, the more likely they were to have an AD. Accordingly, the chances of a patient over 75 years of age having an AD were higher than for a patient aged between 31 and 45 (OR = 0.082, 95% CI 0.015–0.447; *p* = 0.004), 46 and 65 (OR = 0.355, 95% CI 0.169–0.743; *p* = 0.006), or over 65-year-olds (OR = 0.396, 95% CI 0.181–0.868; *p* = 0.021). (Tables 2 and 3) show the results of logistic regression analyses.

**Table 3:** Influencing Factors– Multivariate Logistic Regression Analysis

Variable	N	OR	95% CI	Result of statistical analysis (***p***-value)
Age group (> 75, *n* = 54)	339			
≤ 30	4	0.000	[0.000–0.000]	.
31–45*	**17**	**0.082**	**[0.015–0.447]**	0.004*
46–65*	**188**	**0.355**	**[0.169–0.743]**	0.006*
66–75*	**76**	**0.396**	**[0.181–0.868]**	0.021*
Marital status (Single, *n* = 45)	339			
Married*	**231**	**2.574**	**[1.142–5.802]**	0.023*
Divorced	27	1.562	[0.508–4.799]	0.436
Widowed	26	1.860	[0.593–5.829]	0.287
Permanent partnership*	**10**	**6.900**	**[1.312–36.295]**	0.023*
At least one comorbidity (Yes, *n* = 286)	339	1.413	[0.682–2.930]	0.352
Cardiovascular comorbidity (Yes, *n* = 150)	339	0.843	[0.501–1.421]	0.522
Regular medication (Yes, *n* = 246) *	**330**	**1.896**	**[1.029–3.494]**	0.040*
ECOG performance status (0, *n* = 257)	339			
1	63	0.695	[0.586–1.986]	0.801
2	17	0.698	[0.859–10.442]	0.799
3	2	2.076	[0.073–21.460]	0.641

Reference categories: age group (> 75 years), marital status (single), at least one comorbidity (no), cardiovascular comorbidity (no), regular medication (no), ECOG (stadium 0)

Rows marked with an asterisk (*) indicate the influencing factors that were found to be statistically relevant

Lines marked in bold indicate the influential factors that had proved to be statistically relevant in both univariate and multinomial analyses

*N* Number of patients, *OR* odds ratio, *95% CI* 95% confidence interval, *p* value of significance

Model goodness of fit: −2 Log-likelihood: 61.059; Cox & Snell: 0.091; Nagelkerke: 0.122; McFadden: 0.069

### Further characteristics of advance directives

Patients were asked about both the time of the creation of their ADs and the reasons for, or against, their creation. Of 207 patients with an AD 91 patients (44.0%) had created them before the initial diagnosis of HNC. 51 patients (24.6%) had written their documents with the beginning of the disease. Another 23 patients (11.1%) created their ADs with the knowledge of impending hospitalization. A total of 42 patients did not provide any information in this regard (20.3%).

Regarding the reasons for creating an AD, we received responses from 164 patients (79.2% of patients with ADs). Among them, 26 patients (12.6%) stated that they decided to make one because of positive or negative experiences with medical treatment in the past. 45 patients (21.7%) followed the advice of either a primary care physician, a relative, or a friend. Fear of being abandoned, accompanied by lack of self-determination or medical overtreatment, played a role in 40 patients (19.3%). Only 9 patients (4.3%) had been motivated to prepare an AD by media, public relations or advertising. A further 21.3% (*n* = 44) of patients stated that several reasons were decisive. A total of 43 patients (20.8%) did not answer the question.

Out of 239 patients without ADs, only 2 patients (0.8%) expressed fear of inferior medical care as a reason against creating an AD. 14 patients (5.9%) stated that they did not want to deal with the issue of ADs. 73 patients (30.5%) had never thought about creating an AD. 49.4% (*n* = 118) of patients indicated that they had wanted to look into it but had not done so yet. 20 patients (8.3%) had not answered the question and 10 patients (4.2%) had even given contradictory statements compared to their previous answers.

## Discussion

Our survey shows that less than half of the HNC patients studied had created an AD. DPAHCs alone were present in only 3.6% of these patients, LWs alone in 16.8% and CDs in 26.0%. The presence of a comorbidity or regular medication increased the chances of having an AD, whereas the severity of the HNC or another cancer diagnosis did not play a relevant role. As the age of the patients increased, so did the prevalence of ADs in our cohort. In addition, the social situation played a decisive role, because married patients and patients living in a permanent relationship were more likely to have an AD than single patients.

The frequencies of ADs described in the literature vary widely and show large regional and interdisciplinary differences. In studies of non-cancer patients from the United States of America, 7% of patients admitted for acute cerebral hemorrhage had an appropriate document [17]. In Japan, 44% of patients with various cancers reported having an AD [24]. Kirkpatrick et al. compared 112 cardiology patients with cancer patients (26% vs 31%, *p* = 0.37) from the same hospital and found no significant differences in the frequency of LWs [15]. In a study of 753 cancer patients from China, not a single patient had an AD [18]. Out of 75 American patients referred to an oncology center for therapy, 41% had an LW [20]. Another study showed that only 15% of critically ill patients with non-resectable pancreatic cancer had an LW [19].

In Germany, the situation is similarly variable. In 2002, a representative survey of the German population as a whole showed that only 2.5% of respondents had a living will in 2001 [25]. Three years later, von Oorschot et al. found that although the proportion of living wills in palliative cancer patients was significantly higher than in the general population at 26%, it was still unexpectedly low in the overall patient spectrum [26]. A retrospective study of intensive care patients found that out of 658 patients who died, 12% had an LW. Among the deceased, 105 were cancer patients. A DPAHC was present in only 8% of cases [27]. In 2009, a study on surgical patients showed that LWs existed in 16.7% of cases [28]. The study was repeated in 2019 and this time frequencies of 26.3% for LWs and 15% for DPAHCs were reported, among them 24.4% were cancer patients [29]. In a survey of 503 patients from a haemato-oncology outpatient clinic, the prevalence of LWs was 31% in 2011/2012. More than half of the documents were written after 2009, i.e., after changes had been made to the law on advance healthcare directives [30]. These changes led to an overall increase in the frequency of ADs in Germany, also evident from two representative surveys conducted by the German Hospice and Palliative Association in 2012 and 2017 that show a strong upward trend in available documents from 26 to 43% [13]. In 2017, de Heer et al. reported that in 998 intensive care patients 51.3% of patients stated that they possessed an AD. Present in the patient’s hospital records where only 23% of ADs. Within the entire patient collective 41.3% were cancer patients [14]. None of the above studies specifically addressed HNC patients, so there is no robust evidence on the situation in head and neck oncology to date. The results presented here show that the frequency of 46.4% for ADs in HNC patients is comparable to international and interdisciplinary reports.

A comprehensive overview of the prevalence of ADs in different patient collectives from national and international studies is given in (Table 4).

**Table 4:** Prevalence in advance directives national and internationally

	Prevalence of Advance directives in cancer patients	Prevalence of Advance directives in the general population or in patients with other life-threatening diseases
National (Germany)	- Germany (2011–2012): 503 patients at the hematology and oncology outpatient department of the University Hospital Mannheim: 31% with an advance directiv e[30]	- Germany (2007–2008): 450 general surgical patients: 16.7% with an advance directive [28]
	- Germany (2004): 272 palliative cancer patients: 26% with a LW [26]	- Germany (2002): representative survey of the German population as a whole showed that only 2.5% of respondents had a living will [25]
		- Germany (2009–2010): Medical intensive care unit: 658 patients (16% cancer patients) who died – 12% with an advance directive and 8% with a legal healthcare prox y[27]
		- Germany (2013–214): 998 Intensive care patients in a university hospital: 51.3% stated that they had prepared a document / present in the patient’s hospital record: 23% [14]
		- Germany (2017): 179 general surgical patients (24.4% cancer patients): 26.3% advance directive, 20.7% precautionary power of attorney and 12.3% care directive [29]
		- Germans (general population) with a living will (2017): Since 2012, the proportion has increased from 26 to 43% [13]
International	- USA (2003–2007): The medical records of 1186 consecutive patients with unresectable pancreas cancer were reviewed over a 4-year span: Only 15% had an advance directive in the medical record [19]	- USA (1994 to 1996): 872 patients treated in the ICU: 27% [16]
	- USA (2008–2009): 75 consecutively admitted patients with cancer in the cancer inpatient service: 41% with an advance directive [20]	- USA (2000–2003): 270 non-traumatic intracerebral hemorrhage cases: 7% [17]
	- France (2008–2012): random sample of 197 patients in a hematology department: 64.5% designated a proxy, 6.1% wrote advance directives [23]	- USA (2004): 508 adult ambulatory patients at four academic internal medicine clinical sites at the University at Buffalo: 43.1% of patients claimed to have completed an AD, but of those who said they had, only 25% thought their provider had a copy [31]
	- China (2015): 753 in-patients with cancer in two cancer centers: 0% had an advance directive [18]	- USA (2007): 112 Patients admitted to a cardiac care unit vs. 105 patients on an oncology floor: 26% vs 31% with an advance directive [15]
		- USA (2014): 201 rural Alabama veterans: Only 13% of participants had living wills [32]
		- USA - Systemic Review (2015): Advance directives for older adults in the emergency department (ED): a systematic review: Rates of patient-reported advance directive completion ranged from 21 to 53%, while advance directives were available to ED personnel for 1 to 44% of patients end [33]
		-Korea (2016): Advance directives were completed by just 4.7% of the general adult population [34]
		- Austria (2019): 2285 Australians aged 65 and over accessing health and residential aged care services: approximately half of participants had some form of Advance care planning [35]

Using multivariate regression analysis, we demonstrated “marital status”, “regular medication”, and “increasing age” to be influential factors for ADs in HNC patients.

Zheng et al. investigated factors influencing the preparation of ADs in cancer patients, including 23.2% with HNC. Increasing age, female gender, higher education level, religious affiliation, and higher ECOG status turned out to be significant variables in univariate analysis [14, 18, 19, 23]. While age was a significant factor in our analysis, the other variables were not found to determine the presence of an AD, which may be attributed to differences in the patient populations studied.

Tan et al. also demonstrated increasing age and, contrary to our findings, unmarried marital status as relevant factors influencing the prevalence of ADs in patients with non-resectable pancreatic cancer. In addition, patients who had previously received anticancer therapy had more frequently created ADs [19]. The association between increasing age and the presence of ADs was also shown in a study of veterans without underlying malignancy from a rural area in Alabama and another study of patients with haemato-oncologic disease. Mahaney-Price et al. found religiosity to be a relevant influencing factor in addition to the influence of age [32]. In contrast, religiosity or a specific religion had no significant effect on the decision to create ADs in our patient population.

Looking at the timing of the creation of the ADs, it is noticeable that a large proportion (44%, *n* = 91) were created before the initial diagnosis of HNC. LWs in particular must define a triggering situation and contain clear instructions for action in the event of incapacity to consent. The German Federal Court of Justice has stated on several occasions that LWs are only legally binding if the person concerned has formulated his or her will precisely, has clearly expressed his or her opinion on individual medical measures or on specific illnesses, and the specific situation that has occurred is described there [36–38]. A LW can and should of course be available before the onset of illness, but it is then necessary to adapt it to any changes in the course of the illness. A DPAHC should also always be up to date, but it does not necessarily have to be constantly adapted to the course of the illness. We advocate that patients without ADs should be advised to create both a LW and a DPAHC when they are first diagnosed with advanced HNC. This may be facilitated by suitable specialists around the initiation of treatment. Existing ADs should be discussed with the attending physicians and, if necessary, adapted to the new situation.

A large proportion of patients with ADs have already been motivated to do so by their environment, for example their family doctor or relatives and friends. Advertising and public relations have played only a minor role to date. Therefore, a more systematic approach by the treating physicians should significantly increase both the number and the quality of ADs.

This is also supported by the fact that of 239 patients who had decided against an AD, only 16 patients (6.7%) did so out of a conscious decision. Only 2 patients (0.8%) did so out of concern about inadequate medical care and 14 patients (5.9%) because they did not want to deal with this issue at all. The remaining patients had either never thought about it or had already considered the possibility of creating an AD, but without doing so. The main reasons given in the literature for writing an AD are fear of abandonment and medical overtreatment, and a lack of self-determination. In our patient collective only 19.3% of all patients with an AD stated these as motives [7].

In our clinic, patients are currently not systematically counseled by the attending physician and/or a social worker about the possibility and benefits of an AD. We would like to introduce a more systematic approach in which patients are assisted in preparing ADs upon initial diagnosis of HNC and the health situation should be evaluated regularly, and the documents should be adjusted if necessary.

Data from patients who refused to participate, in accordance with the ethics committee approval, were not collected and are therefore not available. Given that the survey was conducted in the outpatient setting, data from patients whose general condition did not allow them to attend for follow-up or have completed their regular 5-year follow-up are inevitably missing. Furthermore, due to the study setup the prevalence of patients with locoregional recurrence and distant metastatic cancer can be expected to be somewhat reduced. However, ongoing studies are currently addressing especially HNC patients with recurrent and/or metastatic HNC.

Since the questionnaires were only distributed in a quasi-randomized manner, the results must be interpreted accordingly. However, any selection bias that may have arisen is not intentional and cannot be quantified. But the high number of patients, the long study period, and the fact that the entire spectrum of HNC was represented, contribute to a representative patient collective.

Regarding the practicality of ADs in everyday practice, it is important to note that our results are based on patient data from the questionnaires. The actual availability and applicability of ADs were beyond the scope of this study. In this context, de Heer et al. were able to show that there was a large discrepancy between the information given by the patient and the actual availability of the AD, as less than one third of the documents had been handed in at the clinic. The existence of ADs in physical and electronic patient records, combined with their quality (e.g., form of the document, timing and support in drafting, signature, and applicability: situation description, desired actions, etc.), are subjects for future investigation.

Further research on this topic is therefore desirable to raise awareness of this important issue among treating clinicians and patients in order to increase the prevalence, availability and applicability of ADs in clinical practice. This is particularly important for HNC patients, whose disease often takes a crisis-prone course with loss of decision-making ability and capacity to consent. ADs make it possible to treat patients according to their personal ideas and wishes even in the case of incapacity to consent and make it easier for both physicians and relatives to make medical decisions in sometimes ethically difficult situations. Patient autonomy is emphatically expressed, and medical overtreatment is avoided in the best case. Our data offer the potential to identify patients who need additional attention in this regard. Complementary, we are currently conducting studies measuring the symptom burden and palliative care needs in patients with HNC via validated questionnaires to obtain more information about this often-neglected patient population and to further improve ACP in our institution.

## Conclusion

The results show that less than half of HNC patients have an AD. HNC patients that have an AD are mainly of older age, have comorbidities and live in a regulated social setting. The topic is highly relevant and will increasingly confront medical staff because the demand and interest in LWs/DPAHCs will grow due to demographic changes and the rising incidence of HNC. In view of the low prevalence of ADs, great efforts are necessary to support and advise patients in ACP. Awareness should be raised and strengthened on the part of both patients and practitioners through a targeted demand for appropriate documents. This is the first study investigating the current situation in head and neck oncology and providing evidence to identify a patient population in need of appropriate support. Our findings should also serve as a basis to systematically increase the frequency and applicability of ADs and thus optimize the care of HNC patients.

## Supplementary Information

**Additional file 1.**

**Additional file 2.**

**Additional file 3.**

## Data Availability

The datasets used and/or analysed during the current study available from the corresponding author on reasonable request, because the data can not be anonymized.

## Abbreviations

ACP: Advance Care Planning
AD: Advance Directive
CD: Combined directives
CI: 95% Confidence interval
DPAHC: Durable Power of Attorney for Healthcare
HNC: Head and Neck Cancer
LW: Living Will
M: Mean
OR: Odds ratio
SD: Standard deviation
