# Supplementary figures and images for: Advance directives in patients with head and neck cancer - status quo and factors influencing their creation
Source: BMC Palliat Care. 2022 Apr 8;21:47. doi: 10.1186/s12904-022-00932-5 (PMC8991502; doi:10.1186/s12904-022-00932-5)

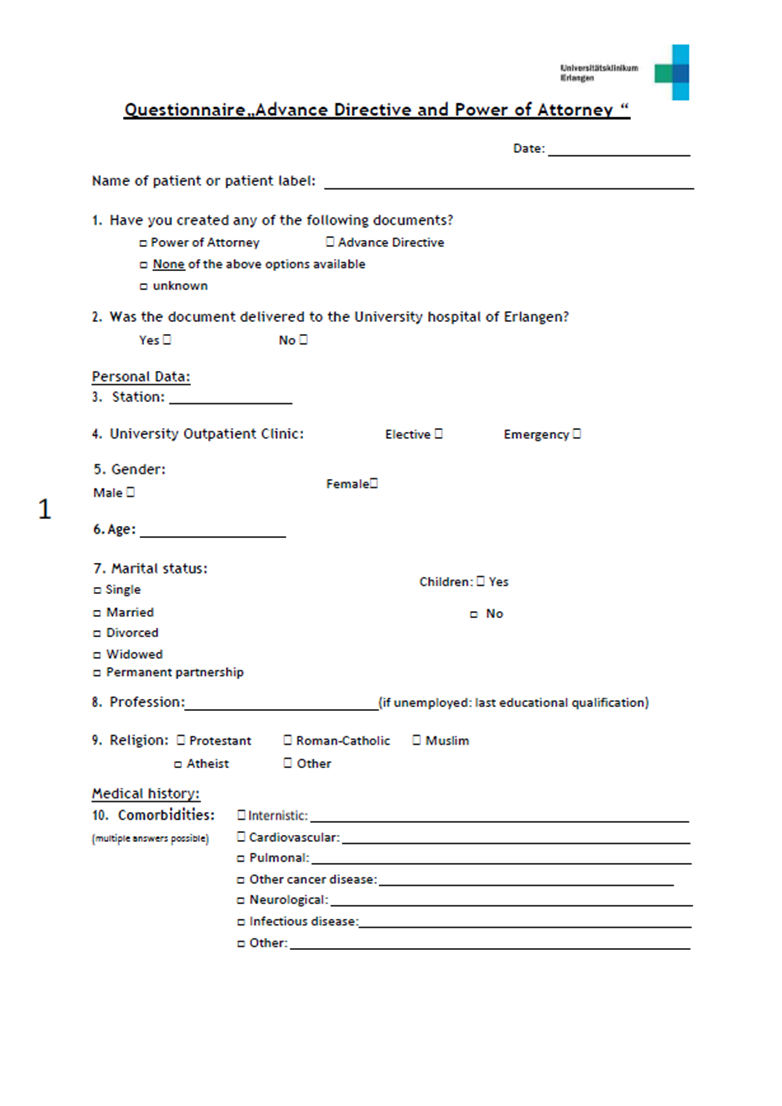

Supplement: Supplementary file 1 — Additional file 1. [file 12904_2022_932_MOESM1_ESM.png]

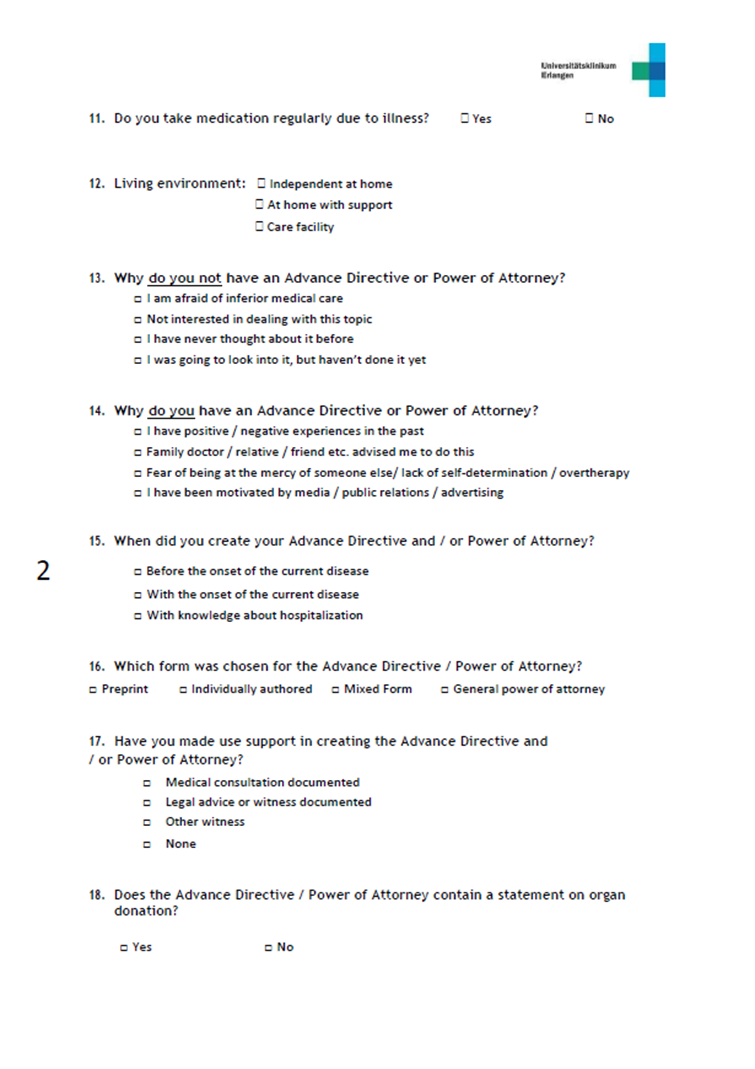

Supplement: Supplementary file 2 — Additional file 2. [file 12904_2022_932_MOESM2_ESM.png]
