# Supplementary material for: Advance directives in patients with head and neck cancer - status quo and factors influencing their creation
Source: BMC Palliat Care. 2022 Apr 8;21:47. doi: 10.1186/s12904-022-00932-5 (PMC8991502; doi:10.1186/s12904-022-00932-5)
Supplement: Supplementary file 3 — Additional file 3. [file 12904_2022_932_MOESM3_ESM.pdf]

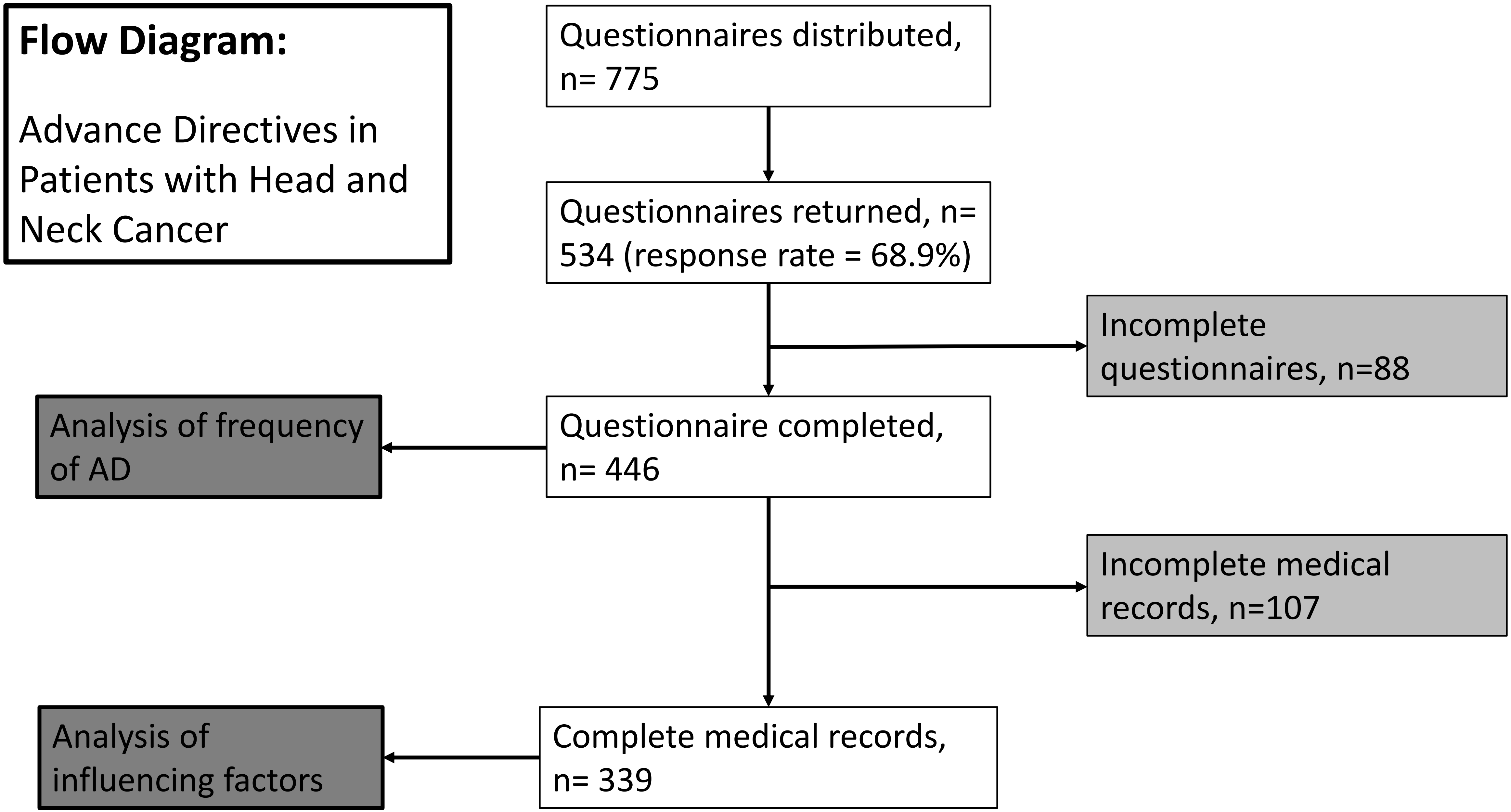
Flow Diagram:
Advance Directives in Patients with Head and Neck Cancer
Questionnaires distributed, n= 775
Questionnaires returned, n= 534 (response rate = 68.9%)
Incomplete questionnaires, n=88
Analysis of frequency of AD
Questionnaire completed, n= 446
Incomplete medical records, n=107
Analysis of influencing factors
Complete medical records, n= 339
